# Antibiotic regimens for neonatal sepsis - a protocol for a systematic review with meta-analysis

**DOI:** 10.1186/s13643-019-1207-1

**Published:** 2019-12-05

**Authors:** Steven Kwasi Korang, Sanam Safi, Christian Gluud, Ulrik Lausten-Thomsen, Janus C. Jakobsen

**Affiliations:** 1 Copenhagen Trial Unit, Centre for Clinical Intervention Research, Department 7812, Copenhagen University Hospital, Rigshospitalet, Copenhagen, Denmark; 2 Department of Neonatology, Copenhagen University Hospital, Rigshospitalet, Copenhagen, Denmark; 3 Department of Regional Health Research, The Faculty of Health Sciences, University of Southern Denmark, Odense, Denmark; 4 Department of Cardiology, Holbæk Hospital, Holbæk, Denmark

**Keywords:** Sepsis, Neonates, Infants, Septic shock, Antibiotics, Systematic review

## Abstract

**Background:**

Sepsis is a major cause of morbidity and mortality among neonates and infants. Antibiotics are a central part of the first line treatment for sepsis in neonatal intensive care units worldwide. However, the evidence on the clinical effects of the commonly used antibiotic regimens for sepsis in neonates remains scarce. This systematic review aims to assess the efficacy and harms of antibiotic regimens for neonatal sepsis.

**Methods:**

Electronic searches will be conducted in MEDLINE, Embase, The Cochrane Library, CINAHL, ZETOC and clinical trial registries (clinicaltrials.gov and ISRCTN). We will include randomised controlled trials of different antibiotic regimens for sepsis of neonates and infants. Eligible interventions will be any antibiotic regimen. Two reviewers will independently screen, select, and extract data. The methodological quality of individual studies will be appraised following Cochrane methodology. Primary outcomes will be ‘all-cause mortality’ and ‘serious adverse events’. Secondary outcomes will be ‘need for respiratory support’, ‘need for circulatory support’, ‘neurodevelopmental impairment’, ototoxicity, nephrotoxicity and necrotizing enterocolitis. We plan to perform a meta-analysis with trial sequential analysis.

**Discussion:**

This is the study protocol for a systematic review on the effects of different antibiotic regimens for neonatal sepsis. The results of this systematic review intent to adequately inform stakeholders or health care professionals in the field of neonatal sepsis, and to aid appropriate development of treatment guidelines.

**Systematic review registration:**

PROSPERO reference number: CRD42019134300.

## Background

### Description of the condition

#### Definition

Sepsis occurring before 28 days after birth is termed neonatal sepsis [1, 2]. There is currently no international consensus on the definition of neonatal sepsis [3, 4]. Most neonatal sepsis criteria used in clinical trials are based on different combinations of clinical and laboratory parameters [4–6].

Due to the lack of consensus on the definition of neonatal sepsis, it is difficult to estimate the exact incidence of neonatal sepsis [1]; however, the incidence is estimated to be between 1 and 12 per 1000 live births in high-income countries [1]. The incidence in low- and middle-income countries is higher, and in Asia, the incidences have been estimated to be up to 38 per 1000 live births [7–12].

Neonatal sepsis is a major cause of morbidity and mortality. It is the third leading cause of neonatal mortality and constitutes 13% of overall, global neonatal mortality [13, 14]. In the high-income countries, neonatal sepsis has a mortality ranging from 5 to 20% and causes major disability (or death) in up to 40% of all cases despite initiation of conventional treatment [1]. Mortality rates up to 70% have been observed in some low- and middle-income countries [1, 4, 15, 16].

In survivors, sepsis is associated with serious long-term morbidity such as cerebral palsy, cognitive and psychomotor delay, auditory and visual impairment, and bronchopulmonary dysplasia [1, 17–19]. Most of these associations are based on observational cohort studies and therefore does not distinguish between causality and association. It remains uncertain whether it is possible to prevent these subsequent sequela by treating neonatal sepsis with appropriate empirical antibiotic regimens [1].

Depending on the time of onset, neonatal sepsis may be divided into early onset sepsis and late onset sepsis. The most commonly accepted distinction between these two subgroups is before and after 72 h (but other definitions, e.g. 48 h and 7 days exist) [1, 2, 20–26]. This distinction is based on the assumed different aetiologies and pathophysiology of pathogens typically seen before and after 72 h [2, 22, 27].

The infection in early onset sepsis is usually acquired vertically from a colonised mother, while the infection in late onset sepsis is usually acquired horizontally, e.g. from the community or a nosocomial (hospital-acquired) infection [16, 22, 26, 28, 29]. However, these theoretical differences might not warrant a need for different antibiotic regimes for early and late onset neonatal sepsis, especially if broad-spectrum antibiotics are used. Accordingly, several trials have included both types of neonates without distinguishing between early and late onset sepsis. As the clinical manifestations also can be non-specific, it can be hard to clinically distinguish between sepsis and deep-seated infections such as meningitis, osteomyelitis and necrotizing enterocolitis [2, 26].

The pathogens causing neonatal sepsis include gram-positive and gram-negative bacteria [30]. The mortality and the distribution pattern of pathogens causing sepsis in neonates differs between low- and middle-income countries and high-income countries. Important pathogen variations can sometimes even be seen between individual *neonatal* intensive care units (NICUs) within a given country. Furthermore, the predominant organisms responsible for neonatal sepsis within regions have also changed with time [31, 32].

Neonates are theoretically immunocompromised as several components of the immune system are not fully developed at birth [2, 33]. This is especially true for preterm newborns, as they are additionally immunocompromised due to an even more immature immune system [34–38]. Prematurity and low birth weight are therefore major risk factors and accordingly, a multi-centre observational study showed that neonatal sepsis were most common in premature (82%) and low birth weight neonates (81%) [39].

Several other risk factors have been shown to be associated with an increased risk of developing neonatal sepsis [21].

For early onset neonatal sepsis, the risk factors are multiple gestation, maternal intrapartum fever, maternal urinary tract infection or chorioamnionitis, prolonged labour, preterm rupture of the membrane (PROM), prolonged PROM > 18 h, and meconium aspiration syndrome [27, 40].

Late onset sepsis also has several risk factors such as mechanical ventilation, intravascular catheterisation, failure of early enteral feeding with breast milk, a prolonged duration of parenteral nutrition, surgery, underlying respiratory and cardiovascular diseases, and hospitalisation [30, 41–44].

### Description of the intervention

Treatment of neonatal sepsis is aimed at treating the underlying infectious cause of sepsis [45, 46], and correcting the associated organic dysfunction through, e.g. respiratory support, circulatory support and correction of metabolic, temperature and glucose derangements [47, 48]. This review assesses the first part, which is to treat the underlying infectious cause of sepsis.

Preliminary results have shown that early initiation of antibiotic therapy in neonates with suspected sepsis seems to reduce both mortality and morbidity [1]. According to guidelines, the treatment should be given as soon as possible and always within 1 h of the decision to treat [49]. The antibiotic therapy is empirical and based on several factors such as age at onset, likely pathogens, and antibiotic susceptibility patterns [21, 31, 50, 51].

Among the most common types of antibiotics used for treatment for neonatal sepsis are beta-lactams (e.g. penicillins, cephalosporins, monobactams and carbapenems), aminoglycosides (e.g. gentamycin) and glycopeptides (e.g. vancomycin and teicoplanin) [52, 53].

The most commonly recommended and used first-line treatment for both early and late onset neonatal sepsis is a beta-lactam antibiotic (most commonly ampicillin, flucloxacillin and penicillin) combined with an aminoglycoside (most commonly gentamicin) [21, 31, 48, 51, 54–57]. However, there has been an increased use of alternative protocols using a cephalosporin (most commonly cefotaxime) or a glycopeptide (most commonly vancomycin) as a first line option to treat especially late onset sepsis [58–60], due to increased resistance among the most common pathogen such as coagulase-negative staphylococci [31, 50]. Ampicillin combined with a third-generation cephalosporin agent (most commonly cefotaxime) is also used as an alternative for early onset sepsis [39, 54, 58–60]. Other regimens such as cephalosporins (as monotherapy) are also used [49]. Guidelines may differ due to local antibiotic resistance of the most common pathogens or whether the empirical regimen is supposed to cover the common but low virulence coagulase-negative staphylococci (for late onset sepsis) [61, 62]. Vancomycin is often considered if staphylococcal infection is suspected [63].

The duration of the antibiotic treatment is adjusted according to the type of pathogen, treatment response, and the possibility of the antibiotic to penetrate to the site of infection in case of, e.g. meningitis, encephalitis, osteomyelitis or endocarditis. A prospective observational study showed that 63% of neonates started in antibiotic therapy were discontinued within 48 h when cultured [58]. When and if a pathogen is identified by cultures, the antibiotic therapy might be changed according to the antibiotic susceptibility of the pathogen. However, causative bacteria are identified only in about one-third of the patients with presumed sepsis [33, 64, 65]. One study found that the empirical antibiotic regimen was changed in 44% of the cases when the pathogen and susceptibility was identified; the most frequently added antibiotics were vancomycin, cefotaxime and penicillin [39]. It is recommended to stop the antibiotic treatment when no signs and symptoms of infection is observed, and no pathogen is identified [2, 55].

#### Antibiotic susceptibility

Antibiotic resistance is a global and growing problem which increases the morbidity, mortality and costs associated with infections [57, 66–68]. The bacterial resistance to antibiotics results mainly from the selective pressure exerted by the use and overuse of antibiotics [67, 69–72]. Studies, comparing antibiotic susceptibility over time in the same unit, show increased resistance to the most used antibiotics [57].

The pathogens causing neonatal infections and their antibiotic susceptibility patterns change over time and may differ among countries [57, 73–77]. When comparing the epidemiology of neonatal sepsis in the low- and middle-income countries with the high-income countries, some important differences emerge in the pattern of etiological pathogens and their antibiotic resistance [11, 78–80].

In high-income countries, data from the UK showed that 95% of the identified pathogens were susceptible to the most commonly used empirical antibiotic regimens of penicillin and gentamicin [54]. In low- and middle-income countries, estimations suggest that up to 70% of pathogens isolated from neonatal sepsis may not be covered by the recommended empirical antibiotic regimen of ampicillin and gentamicin [81]. Some studies in the low- and middle-income countries have shown almost universal antibiotic resistance (92–100% resistant) among the most common pathogens (gram-negative rods) to first-line (often ampicillin and gentamicin) and second-line antibiotics such as the third-generation cephalosporins [15, 48, 81].

In addition, some low- and middle-income countries face widespread dissemination of resistant bacterial strains, including extended-spectrum-lactamase-producing bacteria and methicillin-resistant *Staphylococcus aureus* (MRSA) [81–84].

#### Adverse effects

The use of anti-bacterial agents is potentially associated with adverse effects, but the published data in neonates are scarce and occasionally contradictory.

The use of ampicillin has in some studies been associated with adverse effects such as rashes, diarrhoea, nausea and nephrotoxicity [52, 53, 85]. Contrary to these findings, a recent systematic review of randomised clinical trials showed that ampicillin only increased the incidence of candidiasis with no significant increase in the abovementioned adverse effects [86]. Nephrotoxicity has been estimated to be rare (0.03 %) [85].

Aminoglycosides have been shown to be toxic (nephrotoxic and ototoxic) in adults, whereas its toxicity in neonates remains unclear [87–95].

The most common adverse effects caused by glycopeptides, i.e. vancomycin is fever and phlebitis and in rare cases nephrotoxicity and ototoxicity [96]. There are limited data suggesting a direct causal relationship between toxicity and specific serum vancomycin concentrations [96]. However, in addition to the development of resistance towards vancomycin some observational studies also suggest a three- to fourfold increase in nephrotoxicity when aminoglycosides are combined with vancomycin [96–100].

Cefotaxime is associated with increased risk of death and invasive candidiasis in non-randomised studies [59, 63, 101].

In addition to the specific adverse effects of each antibiotic, extended use of any antibiotics is also associated with higher risk of neonatal candidemia [102, 103].

### How the intervention might work

Antibiotics are antimicrobial drugs that treat and prevent bacterial infections by either killing (bactericidal) or inhibiting the growth of the bacteria (bacteriostatic) [104]. They can be classified based on (1) their mechanism of action (bactericidal or bacteriostatic); (2) bacterial spectrum (broad or narrow); and (3) chemical structure (e.g. penicillins, macrolides, quinolones, tetracyclines or aminoglycosides) [105].

A combination of different antibiotics might have several advantages. Firstly, it is thought to provide an enhanced effect beyond the additive effects of the individual therapies [106]. Secondly, it can be used to broaden the spectrum of antibiotic coverage when used empirically to increase the chance of covering the presumed causative bacteria. Thirdly, a combination therapy is thought to suppress the development of subpopulations of microorganisms resistant to antibiotics [106–108].

However, it is theoretically possible that the optimal empirical antibiotic treatment should not be chosen solely based on the presumed pathogen and cultures. Antibiotics might have different effects in the human body compared with the pattern they show from in vitro (cultures).

### Why it is important to do this review

Despite the high burden of neonatal sepsis, high-quality evidence in diagnosis and treatment is scarce [26]. Yet, in adults, appropriate empirical antibiotic treatment has been shown to halve the fatality associated with sepsis compared with inappropriate empirical antibiotic treatment [109–111].

Due to the diagnostic challenges of sepsis and the relative immunosuppression of the newborn, many neonates receive antibiotics for suspected sepsis. In fact, antibiotics have become the most commonly used pharmacological therapeutic in neonatal intensive care units [112]. Studies suggest that up to 95% of newborns treated with antibiotics for suspected sepsis prove to have no evidence of infection [58, 113, 114]. This presumed overuse of antibiotics seems to contribute to the development and spread of resistant pathogens in the neonatal intensive care units and seems to be associated with adverse events (e.g. invasive candidiasis and increased antimicrobial resistance) [67, 101, 112, 115–117]. Adverse effects of antibiotic exposure in infants is believed to be minimised through the appropriate antibiotic choice and duration of treatment [24].

To create the most appropriate antibiotic policies for neonatal sepsis, there is a need to base these policies on an updated systematic review with meta-analysis.

The latest two Cochrane reviews are from 2004 and 2005 and does not include trials not distinguishing between early and late onset sepsis [118, 119]. Both reviews concluded that there is inadequate evidence from randomised trials in favour of any particular antibiotic regimen for the treatment of suspected early and late onset neonatal sepsis, respectively [118, 119]. No other systematic review has been conducted to assess the effects of different antibiotic regimens for suspected neonatal sepsis regardless of onset.

There is therefore a need for a systematic review to assess the effects of different antibiotic regimens for neonatal sepsis taking into account both risks of systematic errors and random errors [120].

## Objectives

The objective of this study is to compare the beneficial and harmful effects of different antibiotic regimens for neonatal sepsis.

## Methods

The present protocol is being reported in accordance with the reporting guidance the preferred reporting items for systematic reviews and meta-analyses protocols (PRISMA-P) statement [121]. This protocol has been registered within the PROSPERO database (CRD 42019134300).

### Criteria for considering studies for this review

#### Types of studies

Types of studies are randomised clinical trials regardless of publication type, publication status, publication date, and language. We will also include quasi-randomised clinical trials and cluster randomised clinical trials.

#### Types of participants

Neonates and infants suspected of or diagnosed with sepsis (as defined by trialists). We will also include neonates and infants (until 3 months of age) suspected of or diagnosed with severe infections such as meningitis, osteomyelitis, endocarditis and necrotizing enterocolitis.

#### Types of interventions

We will accept any type of antibiotic or combination of antibiotics (regardless of dose and way of administration) such as the following:

1. Beta-lactam antibiotics

- Narrow-spectrum penicillin antibiotics (e.g. oxacillin, cloxacillin, dicloxacillin, nafcillin, methicillin and penicillin G);
- Broad-spectrum penicillin antibiotics (e.g*.* ampicillin, amoxicillin, piperacillin, ticarcillin, carbenicillin and mezlocillin);
- Beta-lactam antibiotics with beta-lactamase inhibitors such as clavulanic acid, sulbactam and tazobactam;
- Cephalosporins (e.g. cefazolin, cephalexin, cefuroxime, cefotetan, cefoxitin, ceftriaxone, cefotaxime, ceftazidime, cefepime, cefazolin, ceftobiprole and cefoperazone);
- Carbapenems (e.g. imipenem, meropenem, doripenem and ertapenem) and monobactams (e.g. aztreonam);

Broad-spectrum penicillins, beta-lactam antibiotics with beta-lactamase inhibitors, cephalosporins and carbapenems will be considered as broad-spectrum antibiotics.

2) Combination of beta-lactam with aminoglycoside (e.g. gentamycin)
3) Combination of beta-lactam with glycopeptide (e.g. vancomycin and teicoplanin)
4) Combination of glycopeptide with aminoglycoside

We plan to assess the following comparisons:

1. Aminoglycoside added to any type of antibiotic versus any type of antibiotic (same antibiotic as in the experimental group).
2. Broad-spectrum beta-lactam antibiotic and aminoglycoside versus narrow-spectrum beta-lactam antibiotic (as defined in the above) and aminoglycoside (same aminoglycoside as in the experimental group).
3. Beta-lactam antibiotic (as defined in the above) and aminoglycoside versus beta-lactam antibiotic and glycopeptide.
4. Any other used antibiotic regimen (not included in the abovementioned comparisons) versus any other used antibiotic regimen (not included in the abovementioned comparisons).

## Co-interventions

We will accept any co-intervention provided they are intended to be delivered similarly to the experimental and the control group. Assuming no interaction, the effects of the co-interventions will ‘even out’ in both groups so the possible effects of antibiotics will be reflected in the results.

We will exclude trials assessing treatment of fungal and viral infections.

### Types of outcome measures

The primary outcomes are as follows:

1. All-cause mortality.
2. Proportion of participants with a serious adverse event defined as any untoward medical occurrence that resulted in death; was life threatening; was persistent or led to significant disability, nephrotoxicity, superinfection, need for respiratory support, need for circulatory support or prolonged hospitalisation [122]. As we expect the trialists’ reporting of serious adverse events to be heterogeneous and not strictly according to the ICH-GCP recommendations, we will include the event as a serious adverse if the trialists either (1) use the term ‘serious adverse event’ but not refer to ICH-GCP or (2) report the proportion of participants with an event we consider fulfil the ICH-GCP definition (e.g. myocardial infarction or hospitalisation). If several of such events are reported then we will choose the highest proportion reported in each trial to avoid double counting.

The secondary outcomes are as follows:

1. Need for respiratory support defined as the need for respiratory support such as non-invasive ventilation (e.g. CPAP) or invasive ventilation (e.g. respirator)
2. Need for circulatory support defined as the need for circulatory support such as fluid bolus or vasoactive medication (e.g. inotropes or vasopressors).
3. Nephrotoxicity (as defined by the trialist).
4. Presence of moderate-to-severe neurological developmental and sensory impairment (defined as a functional abnormality in the function of the brain, spinal cord, muscles, nerves, eyes or ears or as any significant lag in a child’s physical or motor, cognitive, behavioural, emotional or social development, in comparison with other children of the same age and sex within similar environments. If formal evaluation tools were used to assess neurodevelopmental impairment a threshold of − 2 standard deviations of the normal will be used. Furthermore, severe brain injury per se is included, such as intraventricular haemorrhage grade 3 and 4 [123, 124] and periventricular leukomalacia.
5. Ototoxicity (as defined by trialist).
6. Necrotizing enterocolitis during or after treatment, Bells criteria 2 [125].
7. Neurological complication defined as either intraventricular haemorrhage [126], psychomotor retardation, or defined by trialist.

All outcomes will be assessed as proportions.

We will use the trial results reported at maximum follow-up. However, if the trialists report results at multiple time points, we will primarily use the results reported at the time point closest to 1 year.

## Search methods for identification of studies

We will use the criteria and standard methods of Cochrane and Cochrane Neonatal (see the Cochrane Neonatal search strategy for specialized register).

### Electronic searches

We will conduct a comprehensive search including Cochrane Central Register of Controlled Trials (CENTRAL, current issue) in The Cochrane Library; MEDLINE via PubMed (1996 to current); MEDLINE via Ovid (1946 to current) Embase (1980 to current); and CINAHL (1982 to current) using search strategies detailed in Additional file 1.

Further searches will be performed in EMBASE for pharmaceutical publications and ZETOC for abstracts of scientific conferences/symposia. References from identified studies were cross-checked for possible additional studies.

We will search clinical trials registries for ongoing or recently completed trials (clinicaltrials.gov; the World Health Organization’s International Trials Registry and Platform, and the ISRCTN Registry).

We will search all databases from their inception to the present. There will be no restriction by language of publication, and we will arrange for translation where necessary. This will be acknowledged in the ‘Acknowledgements’ section.

### Searching other resources

We will check reference lists of all relevant primary trials and reviews for additional references. To identify unpublished trials, we will also search clinical trial registers of Europe and the USA, websites of pharmaceutical companies, and websites of the US Food and Drug Administration (FDA) and the European Medicines Agency.

## Data collection

### Selection of studies

Two review authors (SKK and SS) will independently screen titles and abstracts. We will retrieve all relevant full-text study reports/publication and two review authors (SKK and SS) will independently screen the full texts and identify trials for inclusion and identify and record reasons for exclusion of the ineligible studies. We will resolve any disagreement through discussion or, if required, by consulting a third person (JCJ). We will record the selection process in sufficient detail to complete a PRISMA flow diagram [127] and “Characteristics of excluded studies” table.

### Data extraction and management

We will use data collection forms for trial characteristics and outcome data which has been piloted on at least one trial in the review. Two review authors (SKK and SS) will extract trial characteristics from included trials. We will extract the following trials characteristics:

1. Methods—trial design, total duration of the trial, number of trial centres and location, trial setting, bias domain items, withdrawals and date of the trial.
2. Participants—number of participants in each intervention group, mean age, age range, sex, diagnostic criteria, inclusion criteria and exclusion criteria.
3. Interventions—intervention and comparison.
4. Outcomes—primary and secondary outcomes specified and collected and time points reported.
5. Notes—funding for trial, and notable conflicts of interest of trial authors.

Two review authors (SKK and SS) will independently extract outcome data from included trials. We will note in the ‘Characteristics of included studies’ table if outcome data were not reported in a usable way. We will resolve disagreements by consensus or by involving a third person (JCJ). We will double-check that data are entered correctly by comparing the data presented in the systematic review with the study reports. A second review author (SS) will spot-check study characteristics for accuracy against the trial report.

### Assessment of risk of bias in included studies

Two review authors will independently assess the risk of bias (low, high or unclear) of all included trials using the Cochrane ‘Risk of bias’ tool [128] for the following domains.

#### Allocation sequence generation

- Low risk: If sequence generation was achieved using computer random number generator or a random numbers table. Drawing lots, tossing a coin, shuffling cards and throwing dice were also considered adequate if performed by an independent adjudicator.
- Unclear risk: If the method of randomisation was not specified but the trial was still presented as being randomised.
- High risk: If the allocation sequence was not randomised or only quasi-randomised.

#### Allocation concealment

- Low risk: If the allocation of patients was performed by a central independent unit, on-site locked computer, identical-looking numbered sealed envelopes, drug bottles or containers prepared by an independent pharmacist or investigator.
- Uncertain risk: If the trial was classified as randomised but the allocation concealment process was not described.
- High risk: If the allocation sequence was familiar to the investigators who assigned participants.

#### Blinding of participants and treatment providers

- Low risk: If the participants and the treatment providers were blinded to intervention allocation, and this was described.
- Uncertain risk: If the procedure of blinding was insufficiently described.
- High risk: If blinding of participants and the treatment providers was not performed.

#### Blinding of outcome assessment

- Low risk of bias: If it was mentioned that outcome assessors were blinded and this was described.
- Uncertain risk of bias: If it was not mentioned if the outcome assessors in the trial were blinded, or the extent of blinding was insufficiently described.
- High risk of bias: If no blinding or incomplete blinding of outcome assessors was performed.

#### Incomplete outcome data

- Low risk of bias: If missing data were unlikely to make treatment effects depart from plausible values. This could either be (1) there were no dropouts or withdrawals for all outcomes or (2) the numbers and reasons for the withdrawals and dropouts for all outcomes were clearly stated and could be described as being similar in both groups. Generally, the trial was judged as at a low risk of bias due to incomplete outcome data if dropouts were less than 5%. However, the 5% cutoff was not definitive.
- Uncertain risk of bias: If there was insufficient information to assess whether missing data were likely to induce bias on the results.
- High risk of bias: If the results were likely to be biased due to missing data either because the pattern of dropouts could be described as being different in the two intervention groups or the trial used improper methods in dealing with the missing data (e.g. last observation carried forward).

#### Selective outcome reporting

- Low risk of bias: If a protocol was published before or at the time the trial was begun and the outcomes specified in the protocol were reported on. If there was no protocol or the protocol was published after the trial was begun, reporting of all-cause mortality and serious adverse events would grant the trial a grade of low risk of bias.
- Uncertain risk of bias: If no protocol was published and the outcomes all-cause mortality and serious adverse events were not reported on.
- High risk of bias: If the outcomes in the protocol were not reported on.

#### For-profit bias

- Low risk of bias: If the trial was not financed by a company that might have an interest in a given result.
- Uncertain risk of bias: If there was no description of how the trial was financed.
- High risk of bias: If the trial was financed or have other involvement by a company that might have an interest in a given result.

#### Other bias

- Low risk of bias: The trial appeared to be free of other bias domains (e.g. academic) that could put it at risk of bias.
- Unclear risk of bias: The trial may or may not have been free of other domains that could put it at risk of bias.
- High risk of bias: There were other factors in the trial that could put it at risk of bias (e.g. authors have conducted trials on the same topic).

#### Overall risk of bias

We assessed overall risk of bias in two groups defined as:

- Low risk of bias: The outcome result was classified as overall ‘low risk of bias’ only if all of the bias domains described in the above paragraphs were classified as low risk of bias.
- High risk of bias: The outcome result was classified ‘high risk of bias’ if any of the bias risk domains described in the above excluding ‘blinding of participants and personnel’ were classified as ‘unclear’ or ‘high risk of bias’.
- Any other bias

Any disagreements will be resolved by discussion or by a third assessor. Our primary conclusions will be based on the results of our primary outcomes at overall low risk of bias. The bias risk assessment enable classification of randomised trials with low risk of bias and high risk of bias. The latter trials tend to overestimate positive intervention effects and underestimate negative effects [129–133].

## Data synthesis

### Measures of treatment effect

#### Dichotomous outcomes

We will calculate risk ratios (RRs) with 95% confidence interval (CI) for dichotomous outcomes.

#### Unit of analysis issues

The unit of analysis will be the participating infant in individually randomised trials and the neonatal unit (or sub-unit) for cluster-randomised trials. For cluster-randomised trials, we will undertake analyses at the level of the individual while accounting for the clustering in the data using the methods recommended in the Cochrane Handbook for Systematic Reviews of Interventions [134].

#### Dealing with missing data

We will not impute missing values for any outcomes in our primary analysis. In two of our sensitivity analyses, we will impute data (see ‘Sensitivity analysis’).

We will contact investigators and trial sponsors in order to verify key trial characteristics and obtain missing numerical outcome data where possible (e.g. when a study is identified as abstract only).

#### Assessment of heterogeneity

We will visually inspect forest plots to assess signs of heterogeneity, and we will explore possible heterogeneity in our prespecified subgroup analyses. We will also inspect trial characteristics across trials to identify clinical heterogeneity. We will assess the presence of statistical heterogeneity by the *χ*^2^ test (threshold *P* < 0.10) and measure the quantities of heterogeneity by the *I*^2^ statistic [135, 136]. If we detect moderate or high heterogeneity, we plan to explore the possible causes (e.g. differences in study design, participants, interventions or completeness of outcome assessments). Ultimately, we may decide that a meta-analysis should be avoided [134].

#### Meta-analysis

We will undertake this meta-analysis according to the recommendations stated in the Cochrane Handbook for Systematic Reviews of Interventions [134]. We will use the statistical software Review Manager 5 [137] provided by Cochrane to analyse data.

We will assess our intervention effects with both random-effects model meta-analyses [138] and fixed-effect model meta-analyses [120, 139]. We will use the more conservative point estimate of the two [120]. We consider ‘the more conservative point estimate’, the estimate closest to zero effect [120]. If the two estimates are equal, we will use the estimate with the widest CI [120].

We will use two primary outcomes, and, therefore, we will consider a *P* value of 0.033 or less as the threshold for statistical significance [120]. For all remaining outcomes, we will consider a *P* value of 0.05 or less as the threshold for statistical significance [120]. We will use the eight-step procedure to assess if the thresholds for significance are crossed [120]. Our primary conclusion will be based on results with low risk of bias [120]. Where data are only available from one trial, we will use Fisher’s exact test [140] for dichotomous data.

Where multiple trial arms are reported in a single trial, we will include only the relevant arms. If two comparisons are combined in the same meta-analysis, we will halve the control group to avoid double counting.

#### Trial sequential analysis

Traditional meta-analysis (TSA) runs the risk of random errors due to sparse data and repetitive testing of accumulating data when updating reviews. We will therefore perform trial sequential analyses on the outcomes, in order to calculate the required information size and the cumulative *Z* curve’s breach of relevant trial sequential monitoring boundaries [141–147]. We wish to control the risks of type I errors and type II errors. A more detailed description of trial sequential analysis can be found at http://www.ctu.dk/tsa/. We will assess our trial sequential analysis intervention effects with both a random effects model [138] and a fixed-effect model [139]. We will use the more conservative point estimate of the two [120]. The more conservative point estimate will be the estimate closest to zero effect. If the two estimates are similar, we will use the estimate with the widest CI.

For dichotomous outcomes, we will estimate the required information size based on the observed, unweighted proportion of patients with an outcome in the control group (the cumulative proportion of patients with an event in the control groups relative to all patients in the control groups), a relative risk reduction of 20%, an alpha of 3.3%, a beta of 20% and diversity as suggested by the trials in the meta-analysis.

### Meta-bias

We will use a funnel plot to assess publication bias if ten or more trials are included. We will visually inspect funnel plots to assess the risk of bias. As we plan to report results when analysing dichotomous outcomes using risk ratios, we will not use any test to assess funnel plot asymmetry when analysing dichotomous outcomes [134].

### Assessment of bias in conducting the systematic review

We will conduct the review according to this published protocol and report any deviations from it in the ‘Differences between protocol and review’ section of the systematic review.

### ‘Summary of findings’ table and GRADE

We will create a ‘Summary of Findings’ table using each of the prespecified primary outcomes and five prespecified secondary outcomes (respiratory failure, circulatory failure, nephrotoxicity, neurological complication and ototoxicity) at maximum follow-up. We will use the Grading of Recommendations Assessment, Development and Evaluation (GRADE) approach, as outlined in the GRADE Handbook [148] to assess the certainty of the body of evidence for our primary outcomes.

Two authors will independently assess the quality of the evidence for each of the outcomes above. We will consider evidence from randomized controlled trials as high quality but downgrade the evidence one level for serious (or two levels for very serious) limitations based upon the following: design (risk of bias), consistency across studies, directness of the evidence, precision of estimates and presence of publication bias. We will assess ‘precision of estimates’ using TSA [120]. We will use the [149] Guideline Development Tool to create a ‘Summary of findings’ table to report the quality of the evidence.

The GRADE approach results in an assessment of the quality of a body of evidence in one of four grades:

1. High: We are very confident that the true effect lies close to that of the estimate of the effect.
2. Moderate: We are moderately confident in the effect estimate: the true effect is likely to be close to the estimate of the effect, but there is a possibility that it is substantially different.
3. Low: Our confidence in the effect estimate is limited: the true effect may be substantially different from the estimate of the effect.
4. Very low: We have very little confidence in the effect estimate: the true effect is likely to be substantially different from the estimate of effect.

### Subgroup analysis and investigation of heterogeneity

We plan to carry out the following subgroup analyses for our primary outcomes.

1. High risk of bias trials compared with low risk of bias trials.
2. Trials assessing neonatal sepsis without separation between early and late onset sepsis compared to separation at either 48 h, 72 h or 7 days.
3. Gestational age: term (≥ 37 weeks) compared with preterm
4. Trials from high-income countries compared with trials from low- and middle-income countries as defined by the World Bank [150]
5. Route of administration such as either oral, intramuscular or intravenous

We will use the formal test for subgroup interactions in Review Manager [137].

### Sensitivity analysis

To assess the potential impact of the missing data, we will perform the two following sensitivity analyses on the primary outcomes.

- ‘Best-worst-case’ scenario: we will assume that all participants lost to follow-up in the experimental group have survived and had no serious adverse event, and all those participants with missing outcomes in the control group have not survived and have had a serious adverse event.
- “Worst-best-case’ scenario. we will assume that all participants lost to follow-up in the experimental group have not survived and have had a serious adverse event and that all those participants lost to follow-up in the control group had survived and had no serious adverse event.

We will present results of both scenarios in our review.

Other post-hoc sensitivity analyses might be warranted if unexpected clinical or statistical heterogeneity is identified during the analysis of the review results [120].

## Discussion

This protocol has several methodological strengths. First, this will be a systematic review that includes neonates with both early and late onset neonatal sepsis, which increases the statistical power and may lead to conclusive results. Second, our methodology is described in detail in this protocol which will be published before the literature search is initiated. Third, we will conduct the review based on the Cochrane Handbook and findings and recommendation of additional methodological studies [120, 135]. Hence, we will systematically assess the risks of systematic errors via bias risk assessments, and we will conduct trial sequential analyses and properly adjust our thresholds for statistical significance to control the risks of random error. This adds further robustness to our results and hence to our conclusions [151]. Forth, we will use our systematic eight step procedure to assess if the thresholds for statistical and clinical significance are crossed [120].

Neonatal sepsis and sepsis among infants are syndromes with high clinical heterogeneity and without internationally agreed upon diagnostic criteria. The underlying bacteria causing sepsis are expected to differ in the different trials as we include trials regardless of onset (in the neonatal period) and location (country). The doses and length of therapy of the antibiotic regimens might also differ between trials, and the trials we will include will possibly use different inclusion criteria. Therefore, the clinical heterogeneity between the trials might be relatively high. This expected heterogeneity may cause significant differences in mortality rates among different trials. In addition, there might be substantial differences in the types of serious adverse events the trials report, which may compromise the validity of the serious adverse event outcome. Also, it is possible that there exist temporospatial differences in other elements of sepsis treatment that may lead to potential differences in results among different trials. We plan to carefully consider these potential limitations in the main publication. We do not expect to include a large number of relevant trials, which potentially will limit the statistical power of this review.

## Supplementary information

**Additional file 1.** Medline via Ovid Search Strategy.

## Abbreviations

MRSA: Methicillin-resistant *Staphylococcus aureus*
NICU: Neonatal intensive care unit
PROM: Preterm rupture of the membrane
