# Supplementary material for: Antibiotic regimens for neonatal sepsis - a protocol for a systematic review with meta-analysis
Source: Syst Rev. 2019 Dec 5;8:306. doi: 10.1186/s13643-019-1207-1 (PMC6896287; doi:10.1186/s13643-019-1207-1)
Supplement: Supplementary file 1 — Additional file 1. Medline via Ovid Search Strategy. [file 13643_2019_1207_MOESM1_ESM.docx]

Medline via Ovid Search Strategy:

| 1 | exp Neonatal Sepsis/ |
| --- | --- |
| 2 | (sepsis adj3 (neonat$ or neo nat$)).ti,ab |
| 3 | (sepsis adj3 (newborn$ or new born$ or newly born$)).ti,ab. |
| 4 | (septic$ adj3 (neonat$ or neo nat$)).ti,ab. |
| 5 | (septic$ adj3 (newborn$ or new born$ or newly born$)).ti,ab. |
| 6 | (infect$ adj3 (neonat$ or neo nat$)).ti,ab. |
| 7 | (infect$ adj3 (newborn$ or new born$ or newly born$)).ti,ab. |
| 8 | (bacter$ adj3 (neonat$ or neo nat$)).ti,ab. |
| 9 | (bacter$ adj3 (newborn$ or new born$ or newly born$)).ti,ab. |
| 10 | (gram adj2 negative).ti,ab. |
| 11 | 1 or 2 or 3 or 4 or 5 or 6 or 7 or 8 or 9 or 10 |
| 12 | exp Anti-Bacterial Agents/ |
| 13 | antibiot$.ti,ab. |
| 14 | antimicrob$.ti,ab. |
| 15 | lactam$.ti,ab. |
| 16 | aminoglycoside$.ti,ab. |
| 17 | glycoprotein.ti,ab. |
| 18 | (penicillin or oxacillin or cloxacillin or dicloxacillin or nafcillin or methicillin).ti,ab. |
| 19 | (ampicillin or amoxicillin or piperacillin or ticarcillin or carbenicillin or mezlocillin).ti,ab. |
| 20 | (cephalosporins or cefazolin or cephalexin or cefuroxime or cefotetan or cefoxitin or ceftriaxone or cefotaxime or ceftazidime or cefepime or cefazolin or ceftobiprole or cefoperazone).ti,ab. |
| 21 | (carbapenems or imipenem or meropenem or doripenem or ertapenem).ti,ab. |
| 22 | (monobactams or aztreonam).ti,ab. |
| 23 | 12 or 13 or 14 or 15 or 16 or 17 or 18 or 19 or 20 or 21 or 22 |
| 24 | 11 and 23 |
| 25 | exp Infant/ |
| 26 | (infan$ or newborn or neonat$ or premature or very low birth weight or low birth weight or VLBW or LBW).mp. |
| 27 | 25 or 26 |
| 28 | 24 and 27 |
| 29 | randomized controlled trial.pt. |
| 30 | controlled clinical trial.pt. |
| 31 | randomized.ab. |
| 32 | placebo.ab. |
| 33 | clinical trials as topic.sh. |
| 34 | randomly.ab. |
| 35 | trial.ti. |
| 36 | 29 or 30 or 31 or 32 or 33 or 34 or 35 |
| 37 | exp animals/ not humans.sh. |
| 38 | 36 not 37 |
| 39 | 28 and 38 |

Pubmed Search Strategy:

(((pubstatusaheadofprint OR publisher[sb] OR pubmednotmedline[sb]))) AND ((((((neonatal sepsis[MeSH Terms]) OR ((sepsis[Title/Abstract] OR septic*[Title/Abstract] OR Infect*[Title/Abstract] OR bacter*[Title/Abstract] OR gram negative[Title/Abstract])))) AND ((AntiBacterial Agents[MeSH Terms]) OR ((antibiot*[Title/Abstract] OR antimicrob*[Title/Abstract] OR lactam*[Title/Abstract] OR aminoglycoside* [Title/Abstract] OR glycoprotein[Title/Abstract] OR penicillin[Title/Abstract] OR oxacillin[Title/Abstract] OR cloxacillin[Title/Abstract] OR dicloxacillin[Title/Abstract] OR nafcillin[Title/Abstract] OR methicillin[Title/Abstract] OR ampicillin[Title/Abstract] OR amoxicillin[Title/Abstract] OR piperacillin[Title/Abstract] OR ticarcillin[Title/Abstract] OR carbenicillin[Title/Abstract] OR mezlocillin[Title/Abstract] OR cephalosporins[Title/Abstract] OR cefazolin[Title/Abstract] OR cephalexin[Title/Abstract] OR cefuroxime[Title/Abstract] OR cefotetan[Title/Abstract] OR cefoxitin[Title/Abstract] OR ceftriaxone[Title/Abstract] OR cefotaxime[Title/Abstract] OR ceftazidime[Title/Abstract] OR cefepime[Title/Abstract] OR cefazolin[Title/Abstract] OR ceftobiprole[Title/Abstract] OR cefoperazone[Title/Abstract] OR carbapenems[Title/Abstract] OR imipenem[Title/Abstract] OR meropenem[Title/Abstract] OR doripenem[Title/Abstract] OR ertapenem[Title/Abstract] OR monobactams[Title/Abstract] OR aztreonam[Title/Abstract]))))) AND (((infant, newborn[MeSH] OR newborn OR neonate OR neonatal OR premature OR low birth weight OR VLBW OR LBW or infan* or neonat*) AND (randomized controlled trial [pt] OR controlled clinical trial [pt] OR randomized [tiab] OR placebo [tiab] OR drug therapy [sh] OR randomly [tiab] OR trial [tiab] OR groups [tiab]) NOT (animals [mh] NOT humans [mh]))))

Embase via Ovid Search Strategy:

| 1 | exp Neonatal Sepsis/ |
| --- | --- |
| 2 | (sepsis adj3 (neonat* or neo nat*)).ti,ab. |
| 3 | (sepsis adj3 (newborn* or new born* or newly born*)).ti,ab. |
| 4 | (septic* adj3 (neonat* or neo nat*)).ti,ab. |
| 5 | (septic* adj3 (newborn* or new born* or newly born*)).ti,ab. |
| 6 | (infect* adj3 (neonat* or neo nat*)).ti,ab. |
| 7 | (infect* adj3 (newborn* or new born* or newly born*)).ti,ab. |
| 8 | (bacter* adj3 (neonat* or neo nat*)).ti,ab. |
| 9 | (bacter* adj3 (newborn* or new born* or newly born*)).ti,ab. |
| 10 | (gram adj2 negative).ti,ab. |
| 11 | 1 or 2 or 3 or 4 or 5 or 6 or 7 or 8 or 9 or 10 |
| 12 | exp Anti-Bacterial Agents/ |
| 13 | antibiot*.ti,ab. |
| 14 | antimicrob*.ti,ab. |
| 15 | lactam*.ti,ab. |
| 16 | aminoglycoside*.ti,ab. |
| 17 | glycoprotein.ti,ab. |
| 18 | (penicillin or oxacillin or cloxacillin or dicloxacillin or nafcillin or methicillin).ti,ab. |
| 19 | (ampicillin or amoxicillin or piperacillin or ticarcillin or carbenicillin or mezlocillin).ti,ab. |
| 20 | (cephalosporins or cefazolin or cephalexin or cefuroxime or cefotetan or cefoxitin or ceftriaxone or cefotaxime or ceftazidime or cefepime or cefazolin or ceftobiprole or cefoperazone).ti,ab. |
| 21 | (carbapenems or imipenem or meropenem or doripenem or ertapenem).ti,ab. |
| 22 | (monobactams or aztreonam).ti,ab. |
| 23 | 12 or 13 or 14 or 15 or 16 or 17 or 18 or 19 or 20 or 21 or 22 |
| 24 | 11 and 23 |
| 25 | (infan* or newborn or neonat* or premature or very low birth weight or low birth weight or VLBW or LBW).mp. [mp=title, abstract, heading word, drug trade name, original title, device manufacturer, drug manufacturer, device trade name, keyword, floating subheading word] |
| 26 | exp infant/ |
| 27 | 25 or 26 |
| 28 | (human not animal).mp. [mp=title, abstract, heading word, drug trade name, original title, device manufacturer, drug manufacturer, device trade name, keyword, floating subheading word] |
| 29 | (randomized controlled trial or controlled clinical trial or randomized or placebo or clinical trials as topic or randomly or trial or clinical trial).mp. [mp=title, abstract, heading word, drug trade name, original title, device manufacturer, drug manufacturer, device trade name, keyword, floating subheading word] |
| 30 | 27 and 28 and 29 |
| 31 | 24 and 30 |

CINAHL Search Strategy:

| S1 | (antibiot* OR antimicrob* OR lactam* OR aminoglycoside* OR glycoprotein OR penicillin OR oxacillin OR cloxacillin OR dicloxacillin OR nafcillin OR methicillin OR ampicillin OR amoxicillin OR piperacillin OR ticarcillin OR carbenicillin OR mezlocillin OR cephalosporins OR cefazolin OR cephalexin OR cefuroxime OR cefotetan OR cefoxitin OR ceftriaxone OR cefotaxime OR ceftazidime OR cefepime OR cefazolin OR ceftobiprole OR cefoperazone OR carbapenems OR imipenem OR meropenem OR doripenem OR ertapenem OR monobactams OR aztreonam) |
| --- | --- |
| S2 | (infan* OR newborn OR neonat* OR premature OR low birth weight OR VLBW OR LBW) AND (randomized controlled trial OR controlled clinical trial OR randomized OR placebo OR clinical trials as topic OR randomly OR trial OR PT clinical trial) |
| S3 | (sepsis N3 (neonat* or neo nat*)) |
| S4 | (sepsis N3 (newborn* or new born* or newly born*)) |
| S5 | (septic* N3 (neonat* or neo nat*)) |
| S6 | (septic* N3 (newborn* or new born* or newly born*)) |
| S7 | (infect* N3 (neonat* or neo nat*)) |
| S8 | (infect* N3 (newborn* or new born* or newly born*)) |
| S9 | (bacter* N3 (neonat* or neo nat*)) |
| S10 | (bacter* N3 (newborn* or new born* or newly born*) |
| S11 | (gram N2 negative) |
| S12 | S3 OR S4 OR S5 OR S6 OR S7 OR S8 OR S9 OR S10 OR S11 |
| S13 | S1 AND S2 AND S12 |

CRS Web Search Strategy:

| 1 | (infan* or newborn or neonat* or premature or preterm or very low birth weight or low birth weight or VLBW or LBW) AND CENTRAL:TARGET |
| --- | --- |
| 2 | MESH DESCRIPTOR Neonatal Sepsis EXPLODE ALL AND CENTRAL:TARGET |
| 3 | (sepsis NEAR3 (neonat* or neo nat*)) AND CENTRAL:TARGET |
| 4 | (sepsis NEAR3 (newborn* or new born* or newly born*)) AND CENTRAL:TARGET |
| 5 | (septic* NEAR3 (neonat* or neo nat*)) AND CENTRAL:TARGET |
| 6 | (septic* NEAR3 (newborn* or new born* or newly born*)) AND CENTRAL:TARGET |
| 7 | (infect* NEAR3 (neonat* or neo nat*)) AND CENTRAL:TARGET |
| 8 | (infect* NEAR3 (newborn* or new born* or newly born*)) AND CENTRAL:TARGET |
| 9 | (bacter* NEAR3 (neonat* or neo nat*)) AND CENTRAL:TARGET |
| 10 | (bacter* NEAR3 (newborn* or new born* or newly born*)) AND CENTRAL:TARGET |
| 11 | (gram NEAR2 negative) AND CENTRAL:TARGET |
| 12 | #2 OR #3 OR #4 OR #5 OR #6 OR #7 OR #8 OR #9 OR #10 OR #11 |
| 13 | MESH DESCRIPTOR Anti-Bacterial Agents EXPLODE ALL AND CENTRAL:TARGET |
| 14 | (antibiot* OR antimicrob* OR lactam* OR aminoglycoside* OR glycoprotein OR penicillin OR oxacillin OR cloxacillin OR dicloxacillin OR nafcillin OR methicillin OR ampicillin OR amoxicillin OR piperacillin OR ticarcillin OR carbenicillin OR mezlocillin OR cephalosporins OR cefazolin OR cephalexin OR cefuroxime OR cefotetan OR cefoxitin OR ceftriaxone OR cefotaxime OR ceftazidime OR cefepime OR cefazolin OR ceftobiprole OR cefoperazone OR carbapenems OR imipenem OR meropenem OR doripenem OR ertapenem OR monobactams OR aztreonam) AND CENTRAL:TARGET |
| 15 | #13 OR #14 |
| 16 | #1 AND #12 AND #15 |
